# Joint Dense Residual and Recurrent Attention Network for DCE-MRI Breast Tumor Segmentation

**DOI:** 10.1155/2022/3470764

**Published:** 2022-04-20

**Authors:** ChuanBo Qin, JingYin Lin, JunYing Zeng, YiKui Zhai, LianFang Tian, ShuTing Peng, Fang Li

**Affiliations:** ^1^Faculty of Intelligent Manufacturing, Wuyi University, Jiangmen 529020, China; ^2^College of Computer Science and Software Engineering, Shenzhen University, Shenzhen 518000, China; ^3^Automation Science and Engineering, South China University of Technology, Guangzhou 510641, China; ^4^Jiangmen Maternal and Child Healthcare Hospital, Jiangmen 529020, China

## Abstract

Breast cancer detection largely relies on imaging characteristics and the ability of clinicians to easily and quickly identify potential lesions. Magnetic resonance imaging (MRI) of breast tumors has recently shown great promise for enabling the automatic identification of breast tumors. Nevertheless, state-of-the-art MRI-based algorithms utilizing deep learning techniques are still limited in their ability to accurately separate tumor and healthy tissue. Therefore, in the current work, we propose an automatic and accurate two-stage U-Net-based segmentation framework for breast tumor detection using dynamic contrast-enhanced MRI (DCE-MRI). This framework was evaluated using T2-weighted MRI data from 160 breast tumor cases, and its performance was compared with that of the standard U-Net model. In the first stage of the proposed framework, a refined U-Net model was utilized to automatically delineate a breast region of interest (ROI) from the surrounding healthy tissue. Importantly, this automatic segmentation step reduced the impact of the background chest tissue on breast tumors' identification. For the second stage, we employed an improved U-Net model that combined a dense residual module based on dilated convolution with a recurrent attention module. This model was used to accurately and automatically segment the tumor tissue from healthy tissue in the breast ROI derived in the previous step. Overall, compared to the U-Net model, the proposed technique exhibited increases in the Dice similarity coefficient, Jaccard similarity, positive predictive value, sensitivity, and Hausdorff distance of 3%, 3%, 3%, 2%, and 16.2, respectively. The proposed model may in the future aid in the clinical diagnosis of breast cancer lesions and help guide individualized patient treatment.

## 1. Introduction

Dynamic contrast-enhanced magnetic resonance imaging (DCE-MRI) is a new functional imaging technique used to assess the physiological properties of the microvascular system in lesions and tissues. This technique is based on the acquisition of baseline images before enhancement as well as consecutive multiperiod high-temporal resolution images after intravenous contrast agent injection. DCE-MRI relies on the calculation of the MRI signal intensity over time and obtains semiquantitative or quantitative parameters that reflect the dynamic enhancement characteristics of the contrast agent in the tissue of interest. In this sense, the perfusion of a lesion is directly represented by the shape of the time-resolved signal intensity curve that represents the dynamic enhancement pattern. Semiquantitative parameters are those derived from the enhancement curve of the tissue for its visual analysis, such as maximum slope, peak value, and area under the curve (AUC). Quantitative parameters refer to contrast-related microcirculatory parameters computed using pharmacokinetic models. Compared with conventional MRI methods, DCE-MRI can not only obtain information about the morphological characteristics of a lesion but also reflect physiological changes in the lesioned tissue.

**Figure 1:**
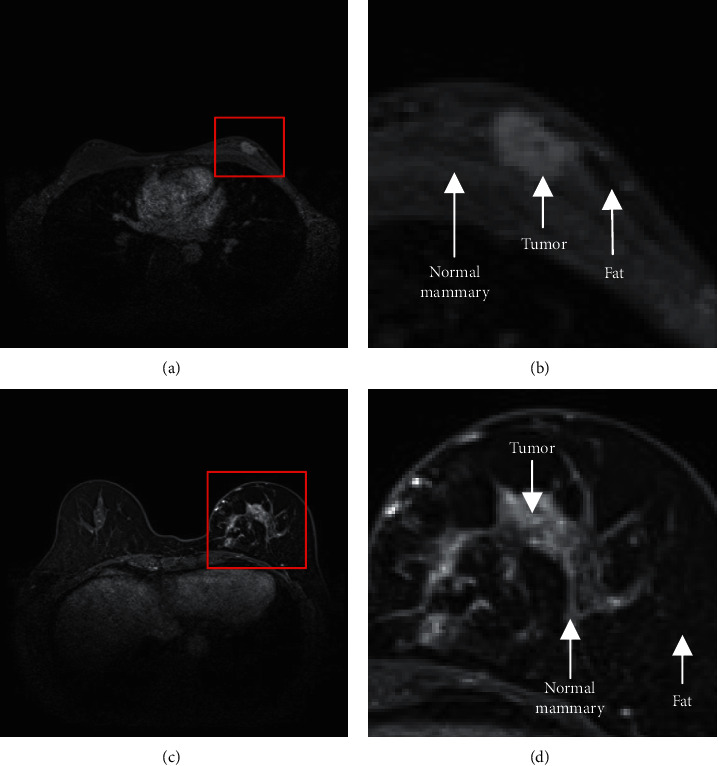
A mass and nonmass DCE-MRI breast tumor. (a) Mass breast tumor. (b) A partially enlarged view of the mass breast tumor. (c) Nonmass breast tumor. (d) A partially enlarged view of the nonmass breast tumor.

Automatic tissue segmentation is paramount for the accurate computer-aided clinical diagnosis of breast cancer using MR-based imaging techniques. Historically, MRI has proven advantageous in detecting breast cancer compared to other imaging methods such as X-ray mammography, as such images effectively provide detailed tissue characteristics that include the invasive range, texture characteristics, structural status, and voxel strength [1]. In particular, DCE-MRI captures one the most detailed human tissue profiles of all MRI-based approaches. With such detailed breast tissue segmentation and characterization, radiologists can infer important additional disease information such as clinical stage and the scope of invasion and structural morphology. This allows clinicians to customize treatment or radiation therapy plans for individual patients and to verify pathological information after surgery. Breast tumors are mainly distributed alongside the mammary glands and manifest as two main subtypes in DCE-MRI images. These two subtypes are generally known as mass type and nonmass type and are shown in Figure 1. The former has clear margins and is fairly straightforward to identify in the images. However, the accurate segmentation of the latter is a challenging task due to the irregular morphology, grayscale heterogeneity, blurred regional boundaries, and low contrast of nonmass tumors. Therefore, the delineation of nonmass breast tumors is in particular a lab-intensive job and requires professional training and extensive clinical experience. This identification is further complicated by the subjectivity of manual demarcation, as well as the psychological fluctuations and physiological tiredness of the reader, which often results in poor repeatability, missed detection, and misjudgment.

Effective breast tumor segmentation results have been achieved using traditional machine learning methods, such as fuzzy c-means (FCM) [2, 3], active contour models (ACM) [4, 5], and Markov random field (MRF) [6, 7]. For example, FCM has recently been utilized to segment 121 breast tumor cases [2]. As noted by Militello et al. [3], the traditional FCM clustering algorithm does not consider any spatial relationship between voxels, making it sensitive to noise and other imaging artifacts. These authors, therefore, used the spatial version of the FCM clustering algorithm for segmentation. Nevertheless, this approach required the a priori selection of an ROI and required the manual adjustment of FCM parameters to achieve the desired segmentation. To reduce these forms of human intervention, a nonlinear dimension reduction scheme termed spectral embedding ACM was proposed [4]. This dimension reduction process is performed on voxels at the same position in different images of DCE-MRI sequences. However, this approach is time intensive when calculating tensor gradients and statistical information. Another study [6] has shown that conditional independence allows loopy belief propagation to condense the multichannel MRF into a single-channel task for tumor segmentation. The disadvantages of this approach are the inability to automatically account for topological changes and the need to manually adjust associated parameters. Support vector machines (SVMs) and independent component analysis (ICA) have also been used to extract data-driven dynamic lesion features [7]. In general, traditional machine learning methods require a priori ROI definition or manual intervention for postprocessing. However, these techniques exhibit poor adaptive generalization and cannot achieve the automatic segmentation of breast tumors.

Recently, the U-Net architecture [8] has been accepted as the common baseline for designing new segmentation models on small-scale datasets [9, 10]. Using the U-Net or similar architectures, significant progress has been achieved in breast tumor segmentation based on mammography, ultrasound, X-ray, and histopathology images [11, 12]. For example, Piantadosi et al. [13] proposed a 3D multiplanar segmentation method with three different U-Net models. Similarly, Wang et al. [14] adopted a mixed 2D and 3D convolution network with multiscale context to compensate for the loss of information when using only 2D networks. In general, the need for such complicated machine learning models stems from the fact that DCE-MRI breast tumor images exhibit a complex and heterogeneous background, which also accounts for only a small proportion of the image. In this sense, the thoracic cavity, breast, muscle, and other tissues occupy a large proportion of the image. This imbalance between the tissue of interest and background activity is a classic problem in machine learning and makes the segmentation task difficult. To address this limitation, a research group at Duke University proposed a mask-guided and hierarchical fully convolutional network (FCN)-based segmentation framework [15–17]. Through the cascaded FCN that utilizes both coarse and fine segmentation models, the breast ROI and detailed segmentation of breast tumors were completed, and it achieved an average Dice similarity coefficient (DSC) of 0.72. Another study [18] proposed an intercascade generative adversarial network (GAN) that contains both an adaptive attention cascade and a joint discrimination network. These components judge whether certain pixels are at the same position. While this approach has its advantages, when compared with FCN, the process of training a GAN is more difficult and its performance is more likely to fluctuate.

There have also been many attempts to improve the U-Net model [19]. The residual U-Net [20] introduced a residual mechanism that can obtain more contextual information and alleviate the degradation of deep networks. Similarly, the dense U-Net [21], which introduced a dense mechanism, extracts richer features without increasing the number of parameters. Oktay et al. [22] also proposed the space-based Attention Gates (AGs), which were integrated with U-Net for pancreas segmentation and exceeded the performance of using only the U-Net. In another study, Rundo et al. [23] merged channel-based Squeeze-and-Excitation (SE) blocks into the U-Net architecture to improve its generalization through adaptive feature re-calibration. Similarly, Guan et al. [24] introduced the SE module into V-Net [25] and achieved excellent performance in a brain tumor segmentation task. By dividing the input image into patches, Chen et al. [26] proposed the TransUNet method by introducing a self-attention mechanism used in natural language processing to the U-Net. Although these attention mechanisms have improved the segmentation performance of the original U-Net model, they all still only use semantic input information. In this regard, we propose a dense residual module and recurrent attention mechanism, which can further improve the segmentation performance of U-Net-based approaches. In the past, multimodal information fusion [27], multicenter [23], context [12], and attention mechanisms have been commonly deployed in end-to-end models to improve segmentation results. Inspired by these previous works, T1-weighted and T2-weighted MR images have also been fused using supervised cross-modal learning. Context and multiscale information have also been fully utilized to construct an automatic end-to-end FCN-based segmentation model [12, 23]. Other research has combined prior shape information obtained by deep learning with ACM to accurately segment breast tumors. However, ACM parameters are not fully trainable and learnable and, in general, generalize poorly.

To address the various drawbacks and limitations of current MR-based breast tumor segmentation techniques, we propose in the current work a two-stage deep learning segmentation framework. This framework incorporates a dense residual module based on dilated convolution [28, 29] as well as a recurrent attention module [22, 30] for adaptive feature map calibration. These components were integrated into the baseline U-Net model to construct the proposed segmentation network termed the Recurrent Attention U-Net. This model was trained and tested on clinical MRI data and exhibited significantly better classification performance compared to the traditional U-Net model. This work may in the future aid in the clinical diagnosis of breast tumors and help guide individualized treatment plans.

## 2. Materials and Methods

### 2.1. Data Acquisition

All breast DCE-MRI images were acquired using a Siemens MAGNETO MESSENZA 1.5 T scanner with a 4-channel phased-array surface breast coil. All patients were in the prone position during image acquisition. T1-weighted fat suppression was utilized to quickly simulate gradient echo. All breast image volumes were acquired in the transverse position with the following parameters: repetition time = 4.6 ms, echo time = 1.7 ms, turning angle = 7°, Field-of-view = 280 × 340 mm^2^, matrix size = 280×340, slice thickness = 1.0 mm, slice gap = 0 mm, and total scan time = 75 s. The contrast agent used was gadopentetate, and the dose was 0.2 ml.

A total of 160 breast tumors cases were included in our clinical database, with 2960 2D T2-weighted slices of size 512 × 512. The images in the axial direction of the transverse plane were utilized for this study. The average length of the DCE-MRI sequences in the database was 112 slices. The number of slices containing breast tumors ranged from 20 to 82 for individual DCE-MRI sequences. The 160 cases were randomly divided into 128 cases for the training set and 32 cases for the test set. Before training, data enhancement operations such as mirroring, scaling, and elastic deformation were performed on the training set. The training set was expanded to 14112 images.

### 2.2. Manual Delineation of Breast ROI

**Figure 2:**
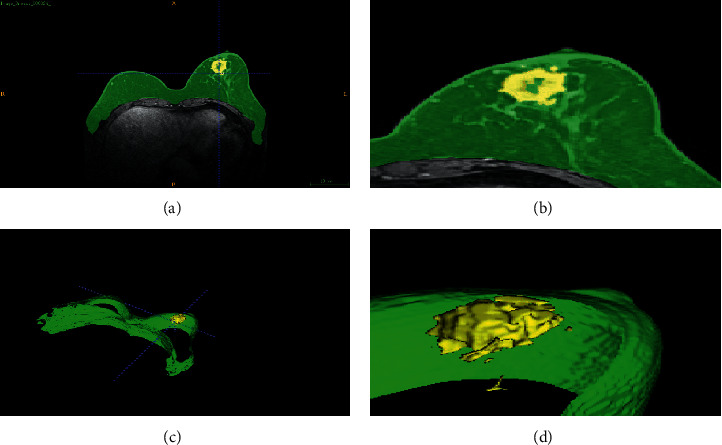
Illustration of DCE-MRI breast ROI and breast tumor. (a) Breast ROI and breast tumor, (b) enlarged breast ROI and breast tumor, (c) 3D reconstruction of breast ROI and breast tumor, and (d) enlarged 3D breast ROI and breast tumor reconstruction. Green and yellow represent breast ROI and breast tumor, respectively.

The ground truth marking of the breast ROI region was performed manually by clinicians with the help of the LabelMe software. The label marking and 3D display of breast tumors was performed using the 3D slice medical imaging software (Figure 2), and the manual delineation of the breast ROI was drawn by clinicians based on their own experience. All breast tumor ROIs were agreed upon by two clinicians and included both mass and nonmass breast tumors.

### 2.3. Breast Tumor Identification

**Figure 3:**
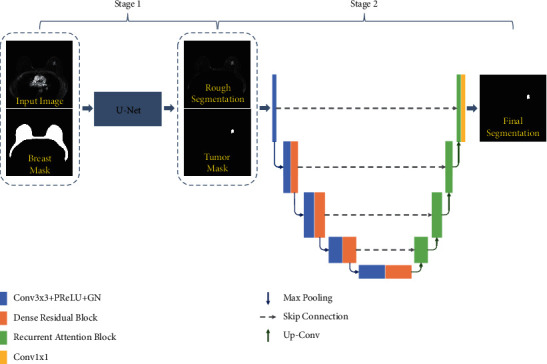
Proposed two-stage breast tumor segmentation framework.

In general, the breast cancer lesion occupies only a small part of the total breast area, which places breast tumor identification into a category of difficult machine learning problems where the classification is largely unbalanced. Hence, advanced and complex segmentation frameworks, usually involving multistage processes, are generally appropriate for this application [15, 16, 30]. In this study, a two-stage deep learning segmentation framework based on FCN was proposed to identify breast cancer lesions from T2-weighted MR images (Figure 3). In stage 1, the classic U-Net architecture was used to complete the automatic segmentation of a breast ROI. In stage 2, a joint dense residual and recurrent attention DCE-MRI breast tumor segmentation network utilizing classic U-Net innovations was employed.

### 2.4. U-Net-Based Segmentation of the Breast ROI

**Figure 4:**
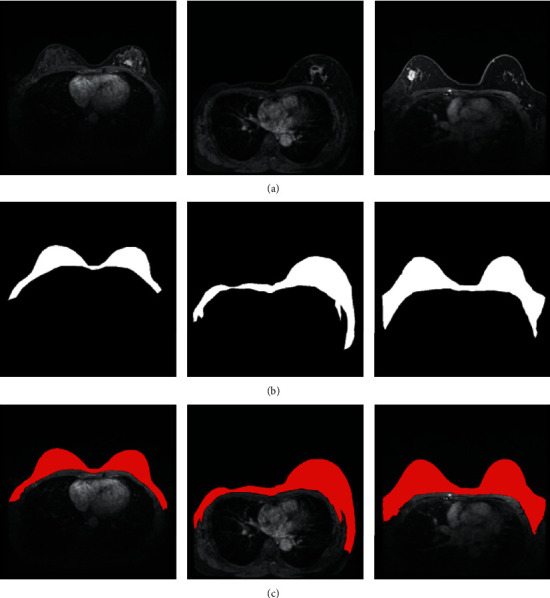
Illustration of breast ROI extraction. The rows represent the original image (a), ground-truth clinician-drawn mask (b), and U-Net-based segmentation results (c).

The breast region ROI was first segmented from the rest of the image, which is important for the later segmentation of tumors. Sketching a rough ROI region of the breast has been shown to effectively remove the influence of other background tissues on the segmentation results [16, 30]. Therefore, we retrained the parameters of the U-Net model based on the breast area that was demarcated by clinicians. By doing so, this model was able to identify a breast ROI that overlapped with clinicians' manual identification remarkably well (Figure 4). In particular, the breast area was extended to both sides of the chest to ensure that no breast tumor area could be missed. It should be noted that both small and large breast sizes could be correctly segmented.

### 2.5. The Proposed Recurrent Attention U-Net Model

**Figure 5:**
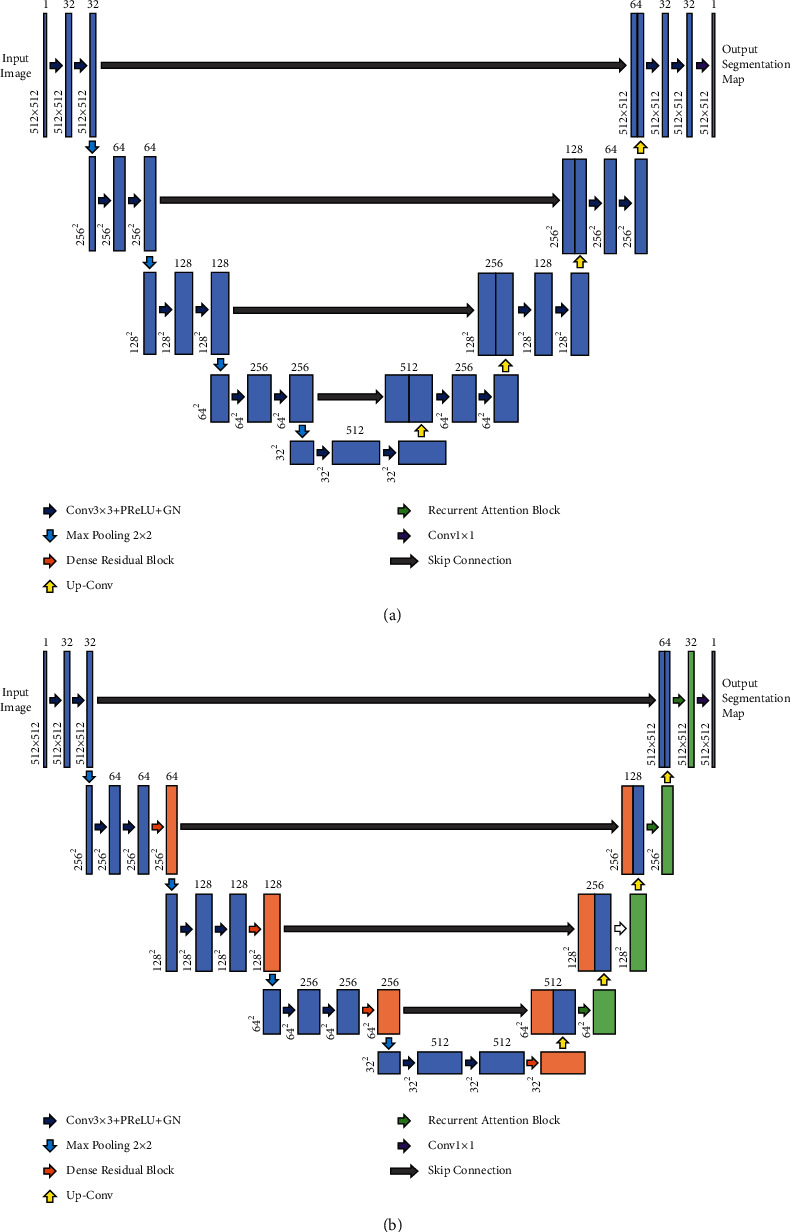
Comparison of the standard U-Net and the proposed recurrent attention U-Net architecture. (a) The standard U-Net consists of a left-right symmetrical encoder and decoder. In the encoder, feature maps are continuously downsampled by max-pooling to extract high-level semantic information. In the decoder, the feature map resolution is gradually recovered by transposed convolution. The rich low-level semantic information of the encoder is concatenated through a skip structure, which compensates for the information loss caused by the downsampling and upsampling process. (b) The proposed recurrent attention U-Net. A dense residual module was added at the end of each stage (except stage 1) of the U-Net encoder, and the output of the module was used for skip connections. Furthermore, our proposed recurrent attention module replaced the convolution blocks at each stage of the U-Net decoder. Note that each rectangle in the picture represents a feature matrix. The number above each rectangle represents the number of feature matrix channels and the number on the lower left represents the resolution of the feature map.

To accurately segment breast tumors from the breast ROI, we propose an end-to-end improved U-Net model, which combines a dense residual module based on dilated convolution with a recurrent attention module. The standard U-Net model and the proposed recurrent attention U-Net model are shown in Figures 5(a) and 5(b), respectively.

#### 2.5.1. Basic Improvement Methods

In the proposed model, we integrated various components to improve the performance of the standard U-Net model. First, all ReLU activation functions were replaced with PReLU [31] activation functions. While PReLU is based on ReLU, it adds learnable parameters to adjust the activation of background noise and irrelevant information. This is in contrast to the ReLU, which instead removes information to prevent the model from learning from noisy inputs. Moreover, the use of the PReLU can simultaneously increase segmentation accuracy and model fitting with negligible extra parameter calculation cost. The second improvement was that all batch normalization in the model was replaced with group normalization (GN) [32]. Third, before each pooling operation, a dense residual module including a dilated convolution operation was added to further expand the receptive field of the model and extract more discriminable feature information. This module output was concatenated with the corresponding input stage in the model decoder to compensate for the feature information lost by downsampling and upsampling. In the decoder, we used the proposed recurrent attention module to replace all the original convolution blocks and utilized a gated recurrent unit (GRU) [33] to combine previous layer information and to extract attention weight several times. Then, we used the attention weight to increase the importance of features so that the model could more accurately locate lesion areas. Other settings of the model were in line with those of a standard U-Net model [8]. Since the size of the breast image was 512 × 512, we reduced the number of output feature channels of all modules in the model to half of that in a standard U-Net model to reduce the computation and memory consumption of the model.

#### 2.5.2. Dense Residual Module

**Figure 6:**
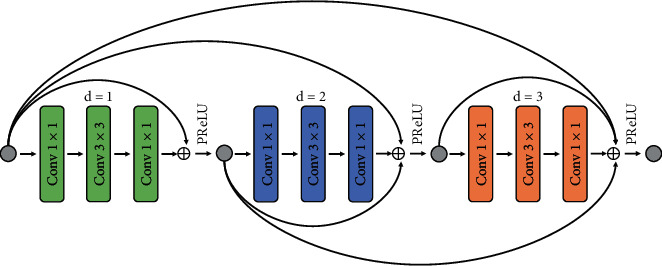
Dense residual module based on dilated convolution. The “Conv1 × 1” represents a convolution block composed of a 1 × 1 kernel size convolution, GN, and PReLU. The “Conv3 × 3” represents a similar block but utilizes a 3 × 3 kernel dilated convolution. The “d” represents the dilation rate. It should be noted that the “Conv1 × 1' after “Conv3 × 3” does not contain a PReLU. To simplify the diagram, this has also been named “Conv1 × 1”.

Based on the residual unit [28], to expand the local learning range of the model, we constructed a new residual unit using dilated convolution to replace the original standard convolution. Then, inspired by the concept of dense connection [11], the output features of the residual unit were passed to all subsequent units for accumulation and summation. This step was meant to realize implicit deep supervision and retain the useful information that was learned. The dense residual module structure is shown in Figure 6. In a residual unit of the module, a standard convolution with a kernel, *k*_*t*_^1^, of size 1 × 1 was used to halve the number of channels, N, of the input feature matrix, *x*_*t*_ ∈ *ℝ*^*W*×*H*×*N*^. Importantly, this caused the width, W, and height, H, to remain unchanged and generated a feature set, *x*_*t*_^1^ ∈ *ℝ*^*W*×*H*×*N*/2^ (N/2 different 1 × 1 × *N* kernel), where *t*=(1,2,3) is the residual unit number. The purpose of this process was to reduce the number of calculations that the model was required to perform. In addition, dilated convolution with the kernel, *k*_*t*_^2^, formed by 3 × 3 kernel size and dilation rate of *d*_*t*_=(1,2,3) was used to enlarge the model's receptive field and to extract the output feature set, *x*_*t*_^2^ ∈ *ℝ*^*W*×*H*×*N*/2^. Finally, the number of feature matrix channels is restored to N by a standard convolution with a kernel, *k*_*t*_^3^, of size 1 × 1, and an output feature set, *x*_*t*_^3^ ∈ *ℝ*^*W*×*H*×*N*^. Except for the last (third) convolutional layer that was followed by a GN [32], the rest of the convolutional layers in the residual unit were followed by a GN and a PReLU [31]. This concept is described as follows:

(1) $$\begin{array}{c} x_{t}^{c}=\left\{\begin{array}{cc} PReLU\left(\text{GN}\left(\sum_{n=1}^{N}k_{t}^{cn}*x_{t}^{c-1n}\right)\right) & \text{if }c\neq 3,\\ \text{GN}\left(\sum_{n=1}^{N}k_{t}^{cn}*x_{t}^{c-1n}\right) & \text{if }c=3, \end{array}\right. \end{array}$$

where *∗* denotes the convolution operation, *c* = (1, 2, 3), and *x*_*t*_^0^=*x*_*t*_. For simplicity, we omitted the bias term. To obtain the final output of the current residual unit, we summed the current residual unit input, *x*_*t*_, and output, *x*_*t*_^3^, as well as all previous unit inputs, (*x*_1_,…, *x*_*t*−1_). After a PReLU activation function, the final feature map, *x*_*t*+1_ ∈ *ℝ*^*W*×*H*×*N*^, was then obtained, as described by


(2) $$\begin{array}{c} x_{t+1}=PReLU\left(x_{1}+\cdots +x_{t-1}+x_{t}+x_{t}^{3}\right). \end{array}$$

In our self-built breast dataset, there were various cases where the lesion areas were very similar to the background. In such cases, automated segmentation can be very difficult. This problem can be alleviated by enhancing the receptive field of the model to obtain richer global context information. There are many ways to enhance the receptive field of a U-Net network. For example, a larger kernel size for convolution or pooling can be used to ensure that the model receives information from a larger receptive field at each pixel and extracts more discriminable features. However, a larger convolution kernel will also greatly increase the computational complexity of the model. Furthermore, the encoding and decoding paths constructed in the standard U-Net are not strictly symmetrical. Although the skip connection and concatenation operations are introduced to ensure that the encoding and decoding processes are consistent in size, the information cannot be guaranteed to be completely reversible. In complex edges and blurred border areas, image information loss is therefore unavoidable. Dilated convolution can alleviate these difficulties to a certain extent and can refine the segmentation results. Dilated convolution can obtain a larger receptive field without changing the size of the convolution kernel and keep feature size unchanged compared to traditional convolution.

Dilated convolution with a dilation rate of *d* and convolution kernel of *k* × *k* was expanded to *k*_*d*_ × *k*_*d*_ by inserting *d* − 1 zeros between each parameter of the convolution kernel. Thus, *k*_*d*_=*k*+(*k* − 1) × (*d* − 1).

However, dilated convolution also has some disadvantages, which are elaborately described in [34]. For a dilated convolution with kernel of size *k*_*d*_ × *k*_*d*_, the effective value used for calculation is only *k* × *k*. When the set dilation rate increases, the proportion of the effective features will decrease. Moreover, the feature information captured by the model will be sparser. To alleviate this issue, we set up three dilated convolutions with dilation rates of (*d*_1_,*d*_2_,*d*_3_)=(1,2,3) within the block. Increasing the dilation rate cascade can fully cover the receptive field, avoid dilation or missing edges in the receptive field, and can solve the problem of information continuity loss.

#### 2.5.3. Recurrent Attention Module

**Figure 7:**
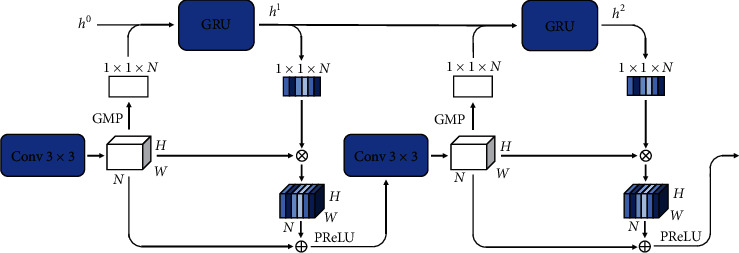
Recurrent attention module. ‘Conv 3×3' represents a convolution block composed of a 3×3 kernel size convolution, GN, and PReLU. ‘GRU' denotes a gated recurrent unit. ‘*h*^0^' is the initial hidden state, and ‘*h*^1^' and ‘*h*^2^' are the output hidden states of GRU. ‘GMP' indicates global max-pooling. See Section 2.5.3 in Section 2.5 of the main text for further explanation.

In the model decoder, each transposed convolution layer is followed by a recurrent attention module, which consists of multiple alternately connected GRUs [33] and convolution blocks (Figure 7), where the convolution block consists of a 3×3 kernel size convolution, a GN, and a PReLU. In each stage of the decoder, the previous stage output is upsampled to the same size as the skip-connected feature map. These two feature maps are then concatenated as the module input. In the module, the first convolution block then halves the number of input feature matrix channels, N, to aggregate features. This procedure is the same as what occurs in the U-Net model, but the subsequent convolution blocks do not change the channel number. A global max-pooling (GMP) is then performed on the output feature map, *x* ∈ *ℝ*^*W*×*H*×*N*^, of the convolution block, to compress its resolution from *H* × *W* to 1 × 1, and extract global information. The output, *x*′∈*ℝ*^1×1×*N*^ , of a GMP process is fed into the GRU together with the hidden state, *h* ∈ ℝ^*N*/2^, which is initialized as a zero vector, and a new hidden state, *h*′∈ℝ^*N*/2^, is extracted according to

(3) $$\begin{array}{c} h_{l}^{\prime }=\text{GRU}\left(\text{GMP}\left(x_{l}^{\prime }\right),h_{l}\right), \end{array}$$

where *l* = (1, 2,…, L) and *L* is the number of GRU. By raising the dimension of *h*′ to be the same as *x*′, we obtained the attention weight, *α* ∈ ℝ^1×1×*N*/2^. Then, we channel-wise multiplied *x* with *α* and added the importance to the feature map, which is an output of the convolution block. In addition, we introduced the residual structure [28], the result of which was followed by a PReLU, according to

(4) $$\begin{array}{c} y_{l}=PReLU\left(x_{l}+\left(x_{l}\times \alpha_{l}\right)\right), \end{array}$$

where *y*_*l*_ ∈ *ℝ*^N×H×*N*^ is the input of the next convolution block.

In Section 2.5.2, we described the addition of the dense residual module to the encoder path, which improved the segmentation accuracy by enlarging the model receptive field. Here, we introduce a recurrent attention mechanism to improve the accuracy of localizing objects and further enhance the segmentation capacity of the model. The general FCN can only learn in a local region with a specific convolution kernel size at any given time to establish implicit and local channel relations for the feature map. Therefore, contextual information outside the local range cannot be recruited. Our proposed recurrent attention module compresses the feature map that is fused with low-level rich semantic information of the dense residual module to a 1×1 resolution by GMP. This realizes the full utilization of all feature information regardless of the size of the convolution kernel. The GRU is the key to gaining attention and exploits the global context of the feature map to extract the attention weights in the channel space. By multiplying the attention weights channel-wise, the explicit channel relationships are constructed for the feature maps, which can guide subsequent convolution layers to learn more efficient representations. Furthermore, attention can improve the sensitivity of the model to the lesion area and enrich the positional information of features from a global perspective. By passing the hidden output state of the GRU, the attention weights are continuously optimized and the feature maps are further calibrated. Altogether, recurrent attention modules can accurately locate breast tumors and better distinguish them from background areas to improve segmentation accuracy.

## 3. Results and Discussion

### 3.1. Training and Evaluation of the Proposed Model

This study was performed on the following platforms: computer server configuration–Intel(R) Core (TM) i9-9900K CPU @ 3.60 GHz × 16, Nvidia GeForce RTX 2080Ti, Linux OS Ubuntu18.04, Programming language–*Python*3.6 and Pytorch1.3 open-source. The initial learning rate was set to 0.001 to train the model. In addition, the standardized initialization function for PReLU presented in [31] was used as the model initializer. Adam was employed with default parameters enhanced by Lookahead [35] as an optimizer to update model parameters to improve convergence speed and the segmentation effect. For all experiments, only one RTX 2080TI graphics card was used to train 200 epochs for each model. In the proposed model, the maximum batch size was set to 4. The calculation results of commonly used batch normalization depend on current batch data. When the batch size is small, the mean and variance of the batch data are poorly represented and thus show a higher error rate. Therefore, the batch normalization originally used by all models was replaced with GN to avoid the influence of small batch sizes on training. Before training, the data were normalized by Z-scores for standard processing to eliminate the influence of different initial gradients on convergence speed.

The number of lesion voxels in our breast dataset was significantly lower than that of nonlesion voxels, which is very common in the field of medical image segmentation. Note that the breast lesion area is generally significantly smaller than the whole breast area. Therefore, the influence of training a model with such an imbalanced dataset on segmentation accuracy is unknown. The loss function of Tversky et al. [36] was used to limit the influence of the imbalanced dataset. Tversky loss is a generalized loss function based on the Tversky index, which can control a better trade-off between precision and recall rate. The formula for this index is represented as

(5) $$\begin{array}{c} T\left(\alpha ,\beta \right)=\frac{\sum_{i=1}^{N}p_{0i}g_{0i}}{\sum_{i=1}^{N}p_{0i}g_{0i}+\alpha \sum_{i=1}^{N}p_{0i}g_{1i}+\beta \sum_{i=1}^{N}p_{1i}g_{0i}}, \end{array}$$

where *p*  and *g* are the network output predictions and corresponding ground truth, respectively. The network output predictions were obtained by mapping the output pixels to probabilities using a soft-max function. Furthermore, *p*_0*i*_ is the probability that voxel at position *i* is a lesion, and *p*_1*i*_  is the probability that voxel at position *i* is a nonlesion. Similarly, *g*_0*i*_ indicates whether the voxel at position *i* is a lesion and *g*_1*i*_ indicates whether the voxel at position *i* is a nonlesion. The values of *α* and *β* control the punishment depth FP_*s*_ and FN_*s*_, respectively. We were able to control the weight between false positives and false negatives by adjusting the hyperparameters *α* and *β*. In the model, we set the values to *α* = 0.3 and *β* = 0.7.

We used segmentation criteria that are standard metrics in the field of medical image segmentation, including area-based Jaccard similarity (Jaccard), dice similarity coefficient (DSC), sensitivity (SEN), specificity (SPE), and positive predictive values (PPV), as well as Hausdorff distance (HD), which can be computed according to equation (6)–(11):

(6) $$\begin{array}{c} \text{Jaccard}=\frac{\text{TP}}{\text{TP}+\text{FN}+\text{FP}}, \end{array}$$

(7) $$\begin{array}{c} \text{DSC}=\frac{2\text{TP}}{2\text{TP}+\text{FP}+\text{FN}}, \end{array}$$

(8) $$\begin{array}{c} \text{SEN}=\frac{\text{TP}}{\text{TP}+\text{FN}}, \end{array}$$

(9) $$\begin{array}{c} \text{SPE}=\frac{\text{TN}}{\text{FP}+\text{TN}}, \end{array}$$

(10) $$\begin{array}{c} \text{PPV}=\frac{\text{TP}}{\text{TP}+\text{FP}}, \end{array}$$

(11) $$\begin{array}{c} \text{HD}=\text{max}\left\{\underset{x\in X}{\max}\underset{y\in Y}{\min}d\left(x,y\right),\underset{y\in Y}{\max}\underset{x\in X}{\min}d\left(x,y\right)\right\}, \end{array}$$

(12) $$\begin{array}{c} \text{Acc}=\frac{\text{TP}+\text{TN}}{\text{TP}+\text{TN}+\text{FN}+\text{FP}}, \end{array}$$

where TP is the true positive rate, FP is the false positive rate, TN is the true negative rate, and FN is the false negative rate. *X* and *Y* represent the pixel set of the input image and ground truth.

### 3.2. Results

In the first stage of the proposed model, we used the retrained U-Net model to segment the breast region ROI from the rest of the image (see Figure 4). The experimental results show that the DSC value reached 0.9198 and the ACC 0.9807 (Table 1), indicating that the model was able to achieve accurate breast ROI segmentation.

**Figure 8:**
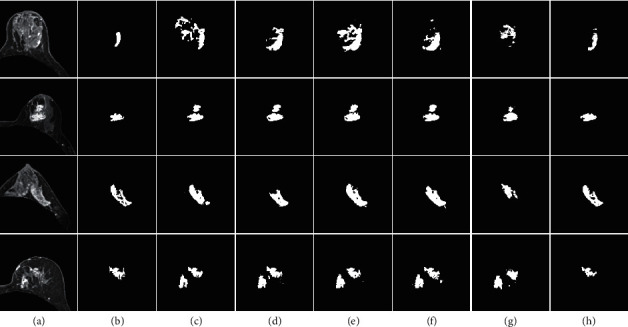
Qualitative analysis of 2D DCE-MRI breast segmentation using both standard and the proposed models. (a) Input image. (b) Ground truth mask. (c) U-Net. (d) Attention U-Net. (e) Residual U-Net. (f) Dense U-Net. (g) V-Net. (h) Present model.

**Figure 9:**
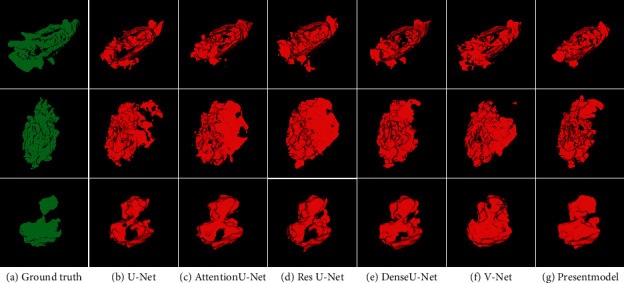
3D reconstruction of the segmentation results for three test cases by the different algorithms.

To evaluate the performance of the present model, we compared the segmentation results with those of the original U-Net model as well as other high-performing medical image segmentation models such as Residual U-Net [8], Dense U-Net [21], Attention U-Net [22], and V-Net [25] on the test set. All compared models were reproduced according to their optimal implementation and utilized the same training and testing protocol with the same experimental dataset.  Figure 8 shows representative segmentation results of four test cases using the aforementioned models. A 3D reconstruction of these segmentation results is shown in Figure 9.

As can be seen in Figure 8, the images in the first and third rows show tumors that are particularly difficult to detect from the surrounding background, often accompanied by an indistinct tumor boundary. When comparing the segmentation results of the comparison models with the ground truth, each model had varying degrees of accuracy in its tumor segmentation results. Nevertheless, the proposed model exhibits a smaller segmentation error than all the comparison models and the segmentation results fit the ground truth boundary better. In contrast, the images in the second and fourth rows show tumors that are extremely similar to the background areas, which are inevitably mis-segmented by the contrast models. However, our model did not only perfectly avoid mislabeling these similar areas, but it also exhibited a more accurate segmentation of the lesion area compared to other models. Therefore, these results suggest that, compared to standard techniques, the recurrent attention U-Net produces more accurate segmentation results and exhibits advantages in lesion edge recognition for tumors of varying sizes and background noise. Figure 9 shows 3D reconstructions of the segmentation of three tumors by the different algorithms. The proposed model segmented these tumors noticeably better than the standard models in terms of surface detail, granularity, hollow processing in the middle, and the edge of the lesion area. Although the recurrent attention U-Net was better in terms of overall error segmentation and missing segmentation performance, there is still a lot of room for improvement compared to the ground truth.

**Figure 10:**
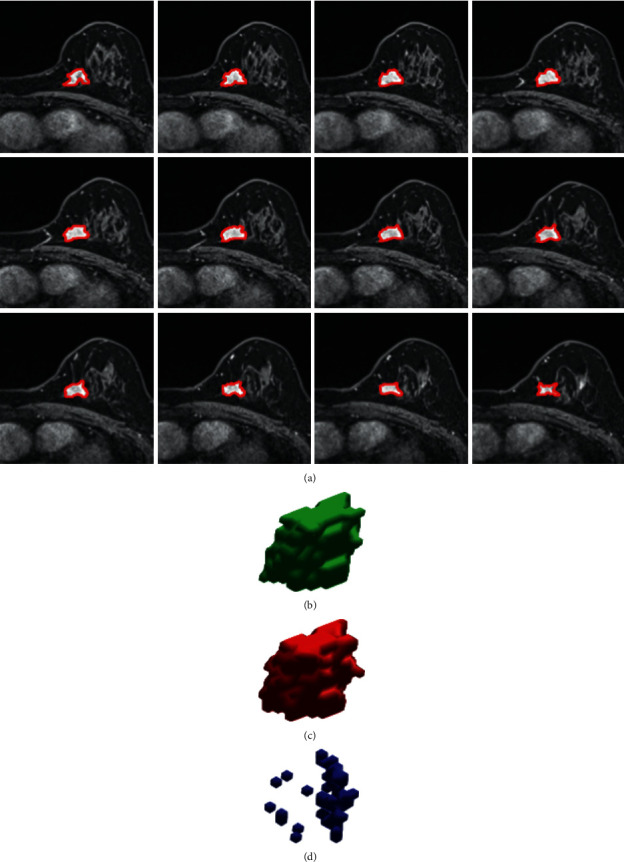
Qualitative analysis of predictive segmentation results of single patient DCE-MRI sequences. (a) A sequence of single patient images with predicted boundaries (red line) and actual areas (white). (b) 3D segmentation result. (c) 3D ground truth. (d) Difference between the ground truth and the predicted segmentation results.

To further evaluate the prediction results of the recurrent attention U-Net for single-patient sequence data, we extracted the boundary of segmentation and fused it with the ground truth image in a 3D reconstruction (Figure 10). The model and ground truth 3D reconstructions exhibited obvious overlap, even at the spatial scale of small tissue branches and fuzzy boundaries (Figure 10(a)). This high performance is likely a result of how the dense residual model improves the model receptive field and how the recurrent attention module makes full use of global context information. The lesion area predicted by the model for a single patient image is shown in Figures 10(b) and 10(c) along with the corresponding ground truth. The difference between the ground truth and the predicted segmentation results is further shown in Figure 10(d). Although from a 2D perspective, the lesion area predicted by the model is highly consistent with the ground truth area, it can be seen from a 3D perspective that there is still room for improvement in terms of pixel-level segmentation accuracy.

In addition to visual inspection, we also quantitatively evaluated the performance of all models on the test set using standard segmentation-based metrics (Table 2). For 32 test samples, we utilized the average values for comparison. Since the breast DCE-MRI clinical dataset used in this study contained noise and suffered from an imbalance in size/pixel number between the background and tumor area, it is expected that any model would exhibit a relatively low SEN and high SPE. Nevertheless, for all evaluation indicators, the performance of the proposed recurrent attention U-Net model surpasses the original U-net. In particular, the proposed model outperformed the standard U-Net model with a 2.7% increase in DSC, a 2.40% increase in PPV, a 3.01% increase in the Jaccard similarity, and an increase in the HD of 16.2. The DSC and Jaccard index is generally the most important metrics for evaluating image segmentation as these two values consider the false-negative rate and false detection rate of pixels in the lesion areas, respectively. Therefore, these metrics best explain the segmentation accuracy of the model comprehensively. Aside from the U-Net model, the proposed model outperformed all other models in all quantitative metrics with the sole exception of SEN, where the Dense U-Net performed slightly better.

**Figure 11:**
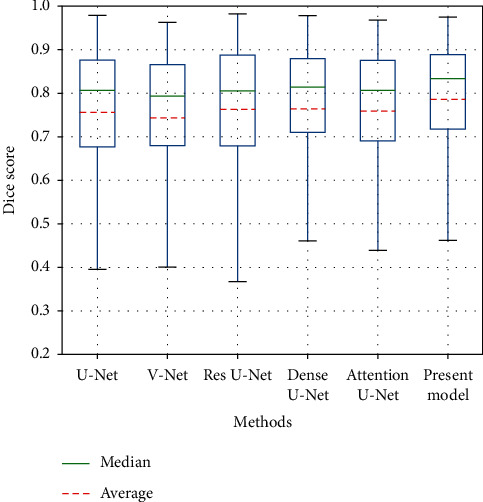
Comparison of experimental results of different models in terms of DSC.

To compare the generalization of each model more graphically, we plotted the DSC index for each model in Figure 11. Notably, compared to other methods, the proposed recurrent attention U-net exhibited the most stable effect. Our model not only outperformed other models in terms of overall performance but also exhibited better performance on target tumors with poorer segmentation effects of other models. This implies that recurrent attention calibration is helpful for U-Net-based segmentation models to locate the lesion area in a complex environment and transfer the focus of the model to the target area.

**Figure 12:**
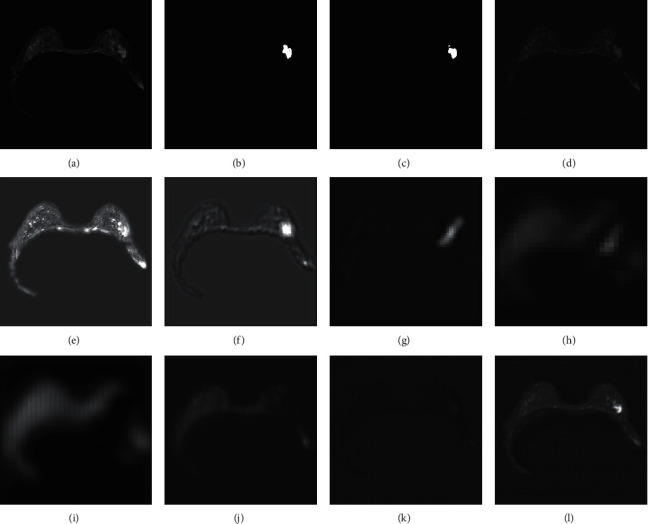
Feature map after fusion of each stage of the proposed model. (a) Input image. (b) Ground truth. (c) Output result. (d) Encoder stage 1. (e) Encoder stage 2. (f) Encoder stage 3. (g) Encoder stage 4. (h) Center stage. (i) Decoder stage 1. (j) Decoder stage 2. (k) Encoder stage 3. (l) Decoder stage 4.

We also investigated the detected features at various stages of the proposed model to gain insight into how this model identified tumor tissue. The output features of each stage of the proposed model were also extracted, as depicted in Figure 12. It can be seen from Figure 12(d)–12(g) that the model encoder started with shallow features such as fine textures and edges and gradually learned more complex semantic information to maintain focus on the breast tumor area.

With a continuous fusion of deep semantic information, the model decoder gradually refined the features of the lesion area. To explain the effectiveness of the proposed module, the weight distribution of the key convolutional layers of the proposed model is visualized and explained, see Figures S1–S3 in the Supplementary Material.

**Figure 13:**
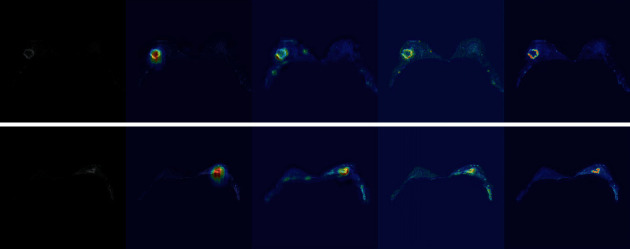
Heat map of each recurrent attention block.

Through the Grad-CAM [37] algorithm, we extracted attention images that have been positioned and calibrated by the recurrent attention module from each layer of the model decoder for visualization.  Figure 13 shows the visualization of two test cases. After the recurrent attention block extracting and applying the attention weights twice, the model located the approximate location of the lesion area and gradually refined the edge to exclude more similar background areas and make the segmentation result more accurate. Therefore, the recurrent attention module helped the model to realize localization and edge refinement of the lesion area, as well as focus attention on the lesion area.

## 4. Conclusion

Automatic segmentation of breast tumors is the key to the fast and early integration of this approach in clinical practice. Due to the segmentation challenges presented by the complex image background of DCE-MRI breast tumors, we proposed in this work a two-stage segmentation framework. In stage 1, FCN was deployed to conduct an automatic delineation of a breast ROI from the background image comprised of the chest and other tissues. In stage 2, we constructed a dense residual module based on the standard U-Net model with a recursive attention module and dilated convolution. For breast tumors of different sizes and shapes in images without postprocessing, a higher segmentation accuracy was achieved by the proposed model compared to other standard U-Net-based models. Therefore, our results showed that the segmentation result of our proposed model improved the DSC by 3%, the Jaccard similarity by 3%, the PPV by 3%, the SEN by 2%, and the Hausdorff distance by 16.2 compared to the standard U-Net model.

Despite these enhancements, there are still some limitations of the current study that should be noted. Although the segmentation accuracy was markedly improved, the values achieved by the proposed models may still not be sufficient for clinical application. The proposed model was only evaluated on a single-source, single-sequence DCE-MRI breast dataset. For multisequence or multicenter studies, its effectiveness has yet to be verified. Furthermore, if the breast area was small or the position of the breast tumor was near the chest cavity, the segmentation may have exhibited additional errors. In addition, this framework is not strictly end-to-end, and in future studies, an in-depth study of the 3D segmentation of breast tumors is needed.

Nevertheless, this study showed that the proposed model can intuitively assist clinicians with observing the position and volume of breast tumors. This work may in the future help clinicians to quantitatively analyze the segmentation results from a 3D perspective and establish a prediction model to improve the diagnosis, radiotherapy, and prognosis of breast tumors [38, 39].

## Figures and Tables

**Table 1:** Segmentation performance of breast ROI identification.

Method	DSC	Jaccard	SEN	SPE	PPV	ACC
U-Net [8]	0.9198	0.8534	0.9289	0.9878	0.9124	0.9807

**Table 2:** Comparison of experimental results of the different prediction models on the test set. All values in this table are averages from 32 test cases.

Method	Jaccard	DSC	SEN	SPE	PPV	HD
U-net [8]	0.6348	0.7557	0.8018	0.9989	0.7688	33.2948
Attention U-Net [22]	0.6386	0.7594	0.8013	0.9990	0.7787	19.5235
Residual U-Net [20]	0.6322	0.7555	0.8220	0.9988	0.7526	25.1890
Dense U-Net [21]	0.6529	0.7667	0.8031	**0.9991**	0.7781	20.9375
V-net [25]	0.6360	0.7587	0.8184	0.9989	0.7559	43.3051
**Present model**	**0.6649**	**0.7827**	**0.8296**	0.9990	**0.7928**	**17.0818**

The best results for the segmentation metrics in the comparison experiment are shown in bold.

## Data Availability

The DCE-MRI breast tumor data used to support the findings of this study have not been made available because of the patients' privacy. The database can be obtained from the corresponding author upon request.

## Supplementary Materials

Supplementary Materials Figure S1(a) illustrates the weight distribution of the model's first convolutional layer. This layer mainly learned rough shallow features, as supported by the widely distributed weights. To make predictions regarding each pixel, the last convolutional layer of the model similarly exhibited a wide but sparse weight distribution, as depicted in Figure S1(b). Similarly, Figure S2 depicts the weight distribution of the convolutional layer before each dense residual block and the weight distribution of the last convolutional layer in each dense residual block. Since the model captured more features under a larger receptive field, each of the distributions shown in Figure S2(b), (d), (f), and (h) are wider than those of Figure S2(a), (c), (e), and (g). Figure S3(b), (d), (f), and (h) show the weight distribution of the convolutional layer after extracting and attaching the attention weights (once) in each recurrent attention block. Compared with the weight distribution before feature calibration shown in Figure S3(a), (c), (e), and (g), feature calibration concentrated the weights. Therefore, these results suggest that recurrent attention mechanisms can help the model to better locate breast tumor areas and capture more effective features.
