# Supplementary material for: Joint Dense Residual and Recurrent Attention Network for DCE-MRI Breast Tumor Segmentation
Source: Comput Intell Neurosci. 2022 Apr 20;2022:3470764. doi: 10.1155/2022/3470764 (PMC9045980; doi:10.1155/2022/3470764)
Supplement: Supplementary Materials — Figure S1(a) illustrates the weight distribution of the model's first convolutional layer. This layer mainly learned rough shallow features, as supported by the widely distributed weights. To make predictions regarding each pixel, the last convolutional layer of the model similarly exhibited a wide but sparse weight distribution, as depicted in Figure S1(b). Similarly, Figure S2 depicts the weight distribution of the convolutional layer before each dense residual block and the weight distribution of the last convolutional layer in each dense residual block. Since the model captured more features under a larger receptive field, each of the distributions shown in Figure S2(b), (d), (f), and (h) are wider than those of Figure S2(a), (c), (e), and (g). Figure S3(b), (d), (f), and (h) show the weight distribution of the convolutional layer after extracting and attaching the attention weights (once) in each recurrent attention block. Compared with the weight distribution before feature calibration shown in Figure S3(a), (c), (e), and (g), feature calibration concentrated the weights. Therefore, these results suggest that recurrent attention mechanisms can help the model to better locate breast tumor areas and capture more effective features. [file 3470764.f1.doc]

## Supplementary Figures

Figure S1. The weight distribution of the first and last convolutional layers of the proposed model.

Figure S2. Convolutional layer weight distribution before the dense residual block and the key convolutional layer weight distribution in the dense residual block. DRB: dense residual block.

Figure S3. The weight distribution of all convolutional layers in each recurrent attention block. RAB: recurrent attention block.
